# Development and implementation of a tool for measuring the training effectiveness of the patient-centered consultation model

**DOI:** 10.12688/mep.17511.2

**Published:** 2022-04-29

**Authors:** Merete Jorgensen, Hanne Thorsen, Volkert Siersma, Christine Winther Bang

**Affiliations:** 1 Center for Education and Research in General Practice, Copenhagen University, Copenhagen, 1014, Denmark

**Keywords:** patient-centered care, medical students, assessment of teaching

## Abstract

**Background:** The patient-centered consultation model comprises four elements: exploring health, illness and disease experiences, understanding the whole person, finding common ground, and enhancing the patient-doctor relationship. This method is taught at the course in general practice at Copenhagen University. The aim of the study was to develop a simple tool consisting of a questionnaire about the patient-centered elements and a test video consultation. The outcome is the change in the students’ ability to identify these elements. Used as a pre-course and post-course test it can inform the teachers which elements of the patient-centered consultation need intensifying in the teaching.

**Methods:** The students from a course in general practice volunteered to participate in all steps of the development. They took part in individual interviews to select items from an already existing questionnaire (DanSCORE). The preliminary questionnaire was tested for face and content validity, pilot-tested and tested for test-retest reliability. All video consultations were transcribed and assessed for patient-centered elements through a conversation analysis. The videos showed medical students seeing real patients.

**Results:** The preliminary version of the questionnaire (called DanOBS) had 23 items. In the subsequent interviews, items were reduced to 17, each with three response options. After a pilot test, the questionnaire was further reduced to 13 items, all strictly relevant to the model and with two response options. The final questionnaire had acceptable test-retest reliability. The number of test consultation videos underwent a reduction from six videos to one.

**Conclusions:** The DanOBS combined with a test video consultation, used as a pre-and post-course test demonstrates for teachers which elements in the patient-centered consultation need to be intensified in the teaching.

## Introduction

The patient-centered consultation model, defined by Levenstein, Brown and Stewart has been taught at the general practice course at Copenhagen University since 1991 ^1– 3^. This model has demonstrated reduced health cost expenses and increased patient satisfaction and compliance ^4, 5^. It was introduced in the sixties among others by Balint ^6^. Later a group of Canadian and English researchers discussed and refined the model ^7^. Medical students express a positive attitude toward the patient-centered model for health care delivery ^8^.

The general practice course takes place just prior to graduation as medical doctors. Here the students work eight days in a general practice clinic seeing patients on their own and video record their consultations. In small group sessions they receive feedback on their videos from peers and a university teacher.

Based on the patient-centered consultation model a simple framework named the “Consultation Process” is used in teaching general practice at Copenhagen University ^9– 12^ (see Table 1).

**Table 1.** The “Consultation Process”.

Make an agreement about the topic for the consultation	The patient’s part (Patient’s narrative)
Clarify the patient`s function	
Clarify the patient`s ideas about symptoms	
Clarify the patient`s feelings	
Clarify the patient`s expectations	
Use summarizing as a mean of obtaining common understanding	
Take history	The doctor’s part (History-taking and examination)
Clinical examination	
Use summarizing as a mean of obtaining common understanding	
Reach common understanding about diagnosis and plan.	The mutual/ common part (Agreement on diagnose and plan)
Inform the patient on how to react to symptoms during the course of the illness (safety-netting)	

Also based on this model a questionnaire (DanSCORE: Danish Structured Observation Registration Evaluation) was developed to be used in a pre-and post-course test. The DanSCORE is completed by students after having watched a test video showing a general practitioner and a simulated patient. Hence, the scoring depends on the video shown. The DanSCORE questionnaire was used in two studies ^13, 14^. Data from the DanSCORE response options were reduced to either “correct” or “incorrect” – one point for correct and zero point for incorrect answer. The outcome was difference in percentage of correct answer before and after course. In the previous two studies, the DanSCORE project demonstrated pronounced differences in the communication items.

Another framework also based on the original patient-centered model is “The Global Communication Consultation Rating Scale” based on the Calgary-Cambridge guide to the medical interview. It has 37 items, each with four response options ^15^. It was developed by general practitioners and used in many medical settings to evaluate communication between healthcare workers and patients or clients ^16– 19^. The rating scale was used in general practice in a small pilot study with 26 students in Sweden in 2019 ^20^. The results were used to investigate medical students’ consultation skills assessed by video recordings of their consultations with real patients. The video recordings were assessed by the students and compared with the ratings of the instructors. The conclusion of the study showed moderate concordance and a need for further research.

The Patient Perception of Patient-Centered Communication (PPCC) for assessing a patient-centered consultation as defined by Levinstein, Brown and Stewart could have been eligible for review, but was meant for patients and clinicians ^21^ and an article about measuring patient-centeredness by Epstein *et al.* did not reveal a questionnaire especially for medical students observing a consultation ^22^.

In 2014 Stewart *et al.* ^23^ reduced the patient-centered consultation model from six to the four most important components:

- exploring health, disease, and illness experiences of the patient
- understanding the whole person
- obtaining common ground
- enhancing the patient-clinician relationship

This model covers the content of the teaching when “The Consultation Process Model” and “Calgary-Cambridge Model” is used. The “Consultation Process Model” is meant to be used in teaching general practice and the Calgary-Cambridge model is to be used anywhere in the health care sector. In the first model, room has been made for the patient’s narrative, which is important as the doctor in general practice must have the whole story as the first health care worker seeing the patient.

The aim of this study was to develop a tool for students to use as self-evaluation and examine the results to guide teaching, both in a concurrent course and in future courses. The tool should consist of a questionnaire and a test video of a consultation in general practice. It was the aim to develop a simple and yet comprehensive questionnaire including the four components described by Stewart *et al.* The test video and the questionnaire should be useful as a pre-and post-course test of the students’ knowledge of the patient-centered consultation model. It should demonstrate for teachers which elements of the patient-centered consultation are successfully taught and which elements need to be intensified, and the effect of the educational interventions.

## Methods

In the development of the questionnaire, which includes three steps, 312 out of 375 (83%) final term general practice students at Copenhagen University participated in autumn 2017 and spring 2018. In the first two steps, 17 students volunteered to an interview. In the third step, 295 (79%) out of 375 possible agreed to participate in the pilot tests.

### First step: Selection of items

It was decided to let students explore and select items containing patient-centered elements from the DanSCORE that they found relevant to the consultation model they are being taught.

In the preliminary interviews in May 2017 two male and two female students, aged 26–30 years, from different small groups, volunteered to be interviewed individually by one of the authors (HT).

The students selected 23 of the 33 DanSCORE items. Five of the ten items they removed were clinical items and five items were about communication in general. In addition, they found six different numbers of response options confusing and the wordings in some items confusing.

The students suggested four response options (”too much”; “appropriate”; “too little”; “not mentioned”) for use in the preliminary version of the questionnaire (now called DanOBS-1), which was then ready for field tests.

### Second step: The field tests

Test for face-and content validity and content coverage was carried out with the first version of the 23-items questionnaire (DanOBS-1). Thirteen students participated in individual interviews (four males and nine females, aged 26 to 30 years). During the interviews, one of the authors (HT) asked the students to read aloud each item and to comment on its relevance to the patient-centered consultation model. They were asked if the items were easy to understand and to complete, and whether the response options were appropriate.

During individual interviews, the number of items were gradually reduced from 23 to 17. Four items were removed. Two items were covered by other items, and two items were irrelevant. New wordings of the items were suggested without distorting the content, and the response options were reduced to three (“yes”; “partly”; “no”).

Finally, both the wordings, the response options and the content of the items were accepted by the students.

### The third step: The pilot tests

In spring 2018, the second 17-items version of the questionnaire (DanOBS-2) was completed by the students after having heard a lecture about the patient-centered consultation model and watched a test video of a student seeing a real patient in general practice. The videos used were face to face consultations and not constructed for the purpose. This procedure was repeated after the five-weeks course with the same students. The students were informed that they were free not to complete the questionnaire.

In addition, four female teachers at the course in general practice at Copenhagen University (aged 42 to 67 years) commented on the preliminary questionnaire. At the first day of the course the students participate in a communication workshop, where they watch and evaluate a consultation video together with two teachers. Here the teachers were asked to fill in the questionnaire. The teachers assessed the order of the items and each item’s relevance to the patient-centered consultation model they taught. Minor changes were made in the order of the questions but not on the items as such.

Taking the results of the pilot tests into account the authors once more thoroughly examined each item and whether a majority of students failed to answer certain questions. As a consequence, some items of the questionnaire were either merged or deleted. The authors also decided to have only two response options (see below).

The questionnaire ended up having thirteen items with each two response options (DanOBS-3).

The steps in the development of the questionnaire are presented in the Zenodo repository. See below.

### Modification of the response options

The students participating in the field test suggested three response options “yes”/“partly”/“no”.

Based on experiences from the pilot test the authors finally decided to use a “yes”/ “no” option forcing the student to decide if a particular element in the model was present or absent. The response option “partly” was often used by the students in the pilot tests and could be an easy way out for students not bothering to decide.

When calculating course effectiveness, the responses will have to be divided into “correct” and “incorrect”. Therefore, the response “partly” was also difficult to handle.

### Reliability of the questionnaire

At a stage where the students had been taught the patient-centered consultation model for three weeks, a test-retest reliability was carried out with the final version of the questionnaire. The students watched the same consultation video and completed the questionnaire twice with an interval of one week where no classes were scheduled. Thirty students took part.

### Implementation of the tool

The tool (the DanOBS questionnaire and the test consultation video) was implemented in the spring term of 2018. After an introduction to the patient-centered consultation model the first day of the course, the students completed the questionnaire after having seen a consultation video with a student seeing a real patient. This was repeated after the course with the same video, in which a female patient presented with rhinosinusitis symptoms.

One point is given for correct answer and zero point for incorrect. These numbers are automatically downloaded into Microsoft Excel and placed in a pre-designed spreadsheet. The results are available immediately after the teaching session and give the teachers information for the next courses. Effect sizes are calculated as the mean difference between the answers before and after the course divided by the mean variation before the course ^24^.

The data are analyzed in Microsoft Excel and R (statistic program).

### Ethics

The study was carried out in accordance with relevant guidelines and regulations. The students were informed that the purpose of the study was the evaluation of the teaching, and they were informed that they could refuse to complete the questionnaires. According to Danish law, studies entirely based on data collected from registers and questionnaires do not need approval from an ethics committee [Government D. Law Nr 593 of 2011.06.14. Act on the ethical treatment of health science research projects; accessed 3rd December 2018) and confirmed by Copenhagen University (registration number 2265044) https://en.nvk.dk/rules-and-guidelines/act-on-research-ethics-review-of-health-research-projects].

The students volunteered to participate in the interviews and field tests. The first author was a teacher at the course, while the interviewer was affiliated to the Department of General Practice as a researcher. None of the other authors participated in the teaching.

The test consultation videos were recorded during the students’ work in general practice. The patients participating are informed verbally and in writing that the videos will be used at the course in general practice at the Copenhagen University. The patients are also informed that the video will be deleted automatically after one year or immediately on request of the patient. The video consultations used in the teaching and presented in the exam are automatically deleted two weeks after the exam. The patients gave written consent. Only students at the actual course have access to test videos on the learning platform and have signed a document to observe professional secrecy. The teachers were general practitioners and had by virtue of that duty of confidentiality.

The students at the course were informed that the data from the questionnaire would be anonymously analysed.

## Results

The new questionnaire with 13 items (DanOBS) corresponds satisfactorily with the four components of the patient-centered consultation model as defined by Moira Stewart in 2014 ^24^:

The first component “exploring illness, health, and disease experiences” is covered by four items describing elements that are new to the students and of importance in a patient-centered consultation.

- *Are the patient’s expectations of the outcome of the consultation clarified? (Item 2)*
- *Are the patient’s ideas about their symptoms clarified? (Item 3)*
- *Is it discussed whether the patient has done anything about their symptoms? (Item 4)*
- *Are the patient’s concerns discussed? (Item 6)*

The second component “understanding the whole person” deals with enough time in the consultation for the patient to tell his/her illness experiences and the effect on his/her daily life.

This element is less complex, easier to assess and covered by two items.

- *Is the impact of the patient’s symptoms on their daily life discussed? (Item 5)*
- *Does the doctor give the patient enough time to talk about their symptoms? (Item 10)*

The third component “finding common ground” is covered by five items. This issue is extremely important in a consultation, especially in general practice, where the doctor might be the only healthcare worker that sees the patient. This issue is focused on in the training and covered by five items.

- *Do the doctor and patient make an agreement on the topics for the consultation? (Item 1)*
- *Does the doctor regularly summarize during the consultation? (Item 7)*
- *Does the doctor make sure that the patient understands the outcome of the consultation? (Item 11)*
- *Is the patient informed about what to react to in the expected course of the illness? (Safety-net) (Item 12)*
- *Does the doctor ensure that the patient understands the rationale for the agreed plan? (Item 13)*

The fourth component: “enhancing the patient-doctor relationship” concerns the doctor’s use of understandable terms, not alienating the patient and the doctor`s use of welcoming body-language. This element is less complex and covered by two items.

- *Does the doctor use term, the patient understands? (Item 8)*
- *Is the doctor’s body language welcoming? (Item 9)*

The new questionnaire was evaluated for relevance and for face and content validity and test re-test reliability. For the results of the reliability test see Table 2.

**Table 2.** Results of the reliability test.

Item	First response ’Correct answers’	Second response ’Correct answers’	Pearson’s correlation coefficient
1	0,77	0,77	1,00
2	0,97	0,93	0,69
3	0,93	0,97	0,69
4	0,77	0,77	1,00
5	0,93	0,93	1,00
6	0,93	0,87	0,68
7	0,90	0,93	0,80
8	0,93	0,93	1,00
9	0,97	0,97	1,00
10	0,80	0,77	0,91
11	0,90	0,90	1,00
12	0,40	0,43	0,80
13	0,73	0,73	1,00

*Pearson’s Correlation Coefficient takes on a value between -1 and +1. Here -1 indicates a perfectly negative linear correlation, 0 indicates no linear correlation between two scores and finally +1 indicates a perfectly positive linear correlation between two scores.*

### Reliability test

In total, 30 medical students watched a test consultation video and answered the DanOBS a week apart with no scheduled teaching lessons in between. A correlation coefficient >70 is acceptable. The correlation coefficients here are regarded as acceptable. Table 2 shows the results of the reliability test.

### Implementation results

The tool (the DanOBS questionnaire and test consultation video) was then implemented in a course in general practice spring 2018 for 59 students (student scores before and after the course for DanOBS can be found in the *Underlying data*). The acceptable percentage of correct answers after the course is targeted to >80% as the students are close to graduation as doctors. Table 3 shows how the spreadsheet of the student’s scores should be interpreted. An example of student scores can be seen in Table 4. In six items the percentage of correct answers after the course is <80%; therefore, teachers at the next course will have to intensify the teaching in these elements.

**Table 3.** General interpretation of the spreadsheet of student scores.

Arrows pointing *upwards* mean that positive learning has taken place but in some cases not enough
Arrows pointing *downwards* mean loss of knowledge or confusion about the topic
Arrows pointing *sidewards* mean no change has taken place
A calculated *effect size >0,20* indicates that the students have learnt about the topic on the course
A calculated *effect size >0,80* indicates that the training on the course had been utmost successful

**Table 4.** An example of data from a spreadsheet from a course in autumn 2018.

No.	Items	Before (%)	After (%)	Diff. (pp)		ES
1	Agenda setting	87,0	97,8	10,9	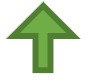	0,32
2	Expectations	69,6	73,9	4,3	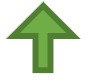	0,13
3	Ideas	91,3	97,8	6,5	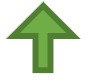	0,19
4	Self-treatment	91,3	97,8	6,5	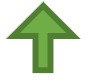	0,19
5	Impact on daily life	84,8	95,7	10,9	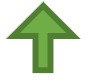	0,32
6	Concerns	87,0	93,5	6,5	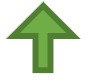	0,19
7	Use of summarising	26,7	51,1	24,4	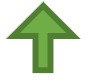	0,72
8	Understandable terms	100,0	97,8	-2,2	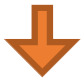	-0,06
9	Body language	65,2	71,7	6,5	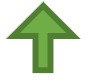	0,19
10	Sufficient time	63,0	65,2	2,2	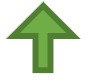	0,06
11	Understand outcome	67,4	63,0	-4,3	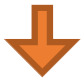	-0,13
12	Safety-net	93,5	100,0	6,5	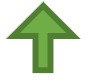	0,19
13	Understand plan	76,1	76,1	0,0	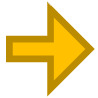	0,00

Diff (pp) means difference in percentage points. See Table 3 for interpretation of the spreadsheet.

## Discussion

An important motivation for the DanOBS was the wish to provide means for the students in the general practice course to self-evaluate their knowledge about the good consultation process. Such evaluation was naturally implemented by pointing out whether the various parts of the “Consultation Process” were present or absent in a video consultation; inquiry into the parts of the consultation process tentatively done by directly asking into them. A preliminary version with 23 items was deemed too cumbersome and had multiple questions asking into the same aspect. Hence, this was brought back to 13 questions adequately and concisely covering all parts of the consultation process, each item addressing a separate aspect of the consultation process. A portfolio of video consultations online now provides for the students’ evaluation needs.

Further motivation for the DanOBS tool was the idea to use the students’ self-evaluation results to guide teaching, both of the concurrent course and future courses. This was implemented by having a common evaluation round at the start and at the end of each course. High percentages of correct answers in certain items in the round at the start of the course would tentatively mean that these aspects may not need much attention in the teaching that follows. Furthermore, if there is not much difference – or even a decline – in correct answers in certain items between start and end of the course, this would mean that teaching in these aspects had not been adequate and should be addressed more in future courses. This was illustrated in Table 4. Hence, DanOBS does not claim to measure teaching quality or tutors’ performance normatively, it merely points towards areas where teaching is particularly needed; primarily aimed at the individual student, but now also made operational for teachers.

Measuring effectiveness by a pre-and post-course test is often used in educational research. Letting students evaluate a consultation as a test is new. Humphris and Kaney, and Baribeau have introduced the OSVE (Objective Structured Video Examen). The students participating in these studies were younger and the consultation models different ^25, 26^. No follow-up has been published.

No instrument has been developed and validated specifically for medical students to complete when observing a consultation. The Global Communication Consultation Rating Scale has been tested for reliability by general practitioners and pilot tested by medical undergraduate students when assessing their own recorded consultation from general practice ^15, 20^. The students evaluated their skills higher than the trained observers.

One way of measuring course effectiveness is to evaluate the students’ performance in an OSCE (Objective Structure Clinical Examination) but most questionnaires or rating scales used are of poor psychometrical quality ^27, 28^. Self-efficacy measurement before and after a course can be used but are often in poor concordance with observed performance ^20, 28^.

Since DanOBS addresses each of the 13 items separately and does not aggregate them into a single (or multiple) score, there is no internal structure to examine. E.g. Cronbach’s alpha measures internal consistency of a sum scale, but we do not sum the items, and while we identify four components of DanOBS, this is mainly to organize teaching and not to create a multidimensional instrument. DanOBS is not meant as a psychometric instrument. 

It is a strength in this study that final year medical students participated in all different steps of the development of the questionnaire. This includes face and content validity, the test-retest reliability and in the pilot tests as well testing the number of videos to be combined with the questionnaire. This is in accordance with Brouwers *et al.*, who state that a questionnaire should be developed in the context where it is going to be used ^29^.

It is a strength that the tool can give immediate information on the effect of educational interventions.

The tool can be used any time during the course to inform the students about their progress in understanding the patient-centered consultation model. If the test can be completed several times and with different videos, it acts as a learning tool.

It could be a limitation that the students volunteered to participate in the testing. For the face and content validity test it was important that the students were enrolled in the course and had the same conceptual understanding of the model. As they were taken away from the teaching session and interviewed, we found it appropriate to let them consent voluntarily.

It could be a limitation that the tool only can be used in students being taught the patient-centered consultation model according to the Calgary-Cambridge model or the Consultation Process model, but these two models are the most widely used in the health care sector. The questionnaire cannot be used by all health care providers. If so, we recommend a new user-specific test for face and content validity.

It is also a limitation that the tool measures a narrow conceptual understanding of the consultation model and cannot be used to measure performance. 

It is a limitation that the pilot test was planned to involve six different videos and ended up with two. In the final stage of the pilot test, one video was used before the course and a different video after the course. It was assumed that a correct answer must be a correct answer no matter which video was shown. However, it could always be questioned whether the two videos were equally easy to assess. Therefore, after the pilot test it was decided in the future to show the same video before and after the course when effectiveness of course was measured.

As the tool detects verbal elements, it can also be used in the assessment of remote video- and telephone consultations

The students in this study observe the verbal expression of patient-centered elements, verified by a conversation analysis by the first author, who has more than 25 years training in evaluating student-patient videos. The DanOBS questionnaire has only two response options “yes”/”no” to ensure that the students are forced to decide if the element is verbally covered. The effect of the course is the change in ability to identify verbal expressions of patient-centered elements from before the course to after the course. To validate the DanOBS questionnaire further would be difficult as no other constructs exists that measure students’ observation of patient-centered elements in a consultation as defined by Stewart *et al.* ^3^

The questionnaire only contains items about patient-centered elements, as the experience from using a more comprehensive questionnaire (DanSCORE) showed most pronounced change in the communication elements that contained most of the patient-centered elements. The students had no problems in evaluating clinical or general issues as they were not new to them. The DanOBS questionnaire resembles to some extent a checklist and the items are evaluated one by one.

Brame found that the attention span of students watching educational videos declines after six minutes ^30^. It was concluded that showing one video with a length of 10–15 minutes is more than enough but can be justified by students being eager to learn what is expected of them in the final exam.

## Conclusion

A simple and short questionnaire combined with one test video for measuring the effect of teaching patient-centered consultation to medical students has been developed and presented in a Microsoft Excel spreadsheet to inform the teachers about the effectiveness of their teaching.

## Practice implication

A simple tool for teachers to assess the effectiveness of training patient-centered consultations has been developed, called DanOBS. The final questionnaire with 13 items is short and easy for students to complete after having watched a consultation video.

The tool can be used to measure the effect of teaching and of interventions (workshops, role-playing etc.) as it is simple, does not take up much time in a time-pressured study program, and gives immediate responses. The tool can be used during the course as supplementary learning material

## Data availability

### Underlying data

Zenodo: Development and implementation of a tool for measuring the training effectiveness of the patient-centered consultation model, https://doi.org/10.5281/zenodo.6477458 ^31^.

 This repository contains the following underlying data:

- Reliability calculation.xlsx (full scores from the reliability test)

### Extended data

Zenodo: Development and implementation of a tool for measuring the training effectiveness of the patient-centered consultation model, https://doi.org/10.5281/zenodo.6477458 ^31^.

This repository contains the following extended data:

- DanOBS_questionnaire.docx (questionnaire)
- Example of use of spreadsheet.xlsx (an example of the use of the tool. Data from one course._2.xlsx (scores before and after course))
- Empty_spreadshet.xlsx (Microsoft Excel scoring spreadsheet (blank copy))
