# Supplementary material for: Development and implementation of a tool for measuring the training effectiveness of the patient-centered consultation model
Source: MedEdPublish (2016). 2022 Apr 29;12:18. Originally published 2022 Mar 14. [Version 2] doi: 10.12688/mep.17511.2 (PMC9427081; doi:10.12688/mep.17511.2)
Supplement: Peer review history

# Peer review: Reviewer response for version 2

**DOI:** 10.21956/mep.20511.r32435

**Published:** 2022-08-11

**Authors:** Asmaa F. Sharif

**Affiliations:** 1 Clinical Medical Sciences Department, College of Medicine, Dar Al Uloom University, Riyadh, Saudi Arabia

The authors proposed a modified form of (DanSCORE) questionnaire as a means of improving the student’s perception of the patient-centered consultation model. The idea seems interesting, and I have a few comments that might improve the manuscript:

- I suggest the authors could add a detailed description to the general practice course in terms of the weight of eight days to the curriculum, and the conventional evaluation method. Were the conventional grades of the course pass or fail or was it graded out of total mark? Besides, and as I could understand, the authors change the formatting of the evaluation method. As they mentioned the old framework adopted peer evaluation, while in the proposed model, the authors talked about a self-evaluation form. The reader might get benefit from more clarification concerning keeping the peer evaluation side to side with self-evaluation or omitting it.
- The main concern about the idea of the work comes from the sole subjective nature of the evaluation. Students might think that they are well skilled and of high level. It would be more reliable and objective if the authors compared the grades and achievements of this cohort of students with controls or with other batches enrolled in classical courses. OSCE exam might be a suitable tool to assess students’ performances. Tutor evaluation is another suggested evaluation form.
- Moreover, I would expect the authors to clarify if students were allowed to watch only a specific category of patients (i.e. outpatients or cold cases) or if students can see all types of patients. On which basis the used videos were selected? If there are any eligibility criteria for videos to be included, I suggest adding them to a flowchart.
- The authors mentioned response rates of 79-83%. What about the remaining 17% of candidates? why do the authors exclude them? What about students disinterested in participating? Elaborate more.
- The authors should give an explanation about changing the response from Yes, No, and Partly to Yes and No in spite of the previous agreements of students in step 2. Add a reference supporting your point of view about the difficulty in handling the response partly.
- It is fine that the authors calculated the reliability of the used tool, I was expecting them also to mention the value of Cronbach alpha to estimate the validity of the used questionnaire. Though I could read the author's argument about the non-psychometric nature of the adopted questionnaire, I recommend calculating the validity for the four components separately.
- Add the version of the software (Microsoft Excel and R) used in statistical analysis.

Is the rationale for developing the new method (or application) clearly explained?

Yes

Is the description of the method technically sound?

Partly

Are the conclusions about the method and its performance adequately supported by the findings presented in the article?

Partly

If any results are presented, are all the source data underlying the results available to ensure full reproducibility?

Yes

Are sufficient details provided to allow replication of the method development and its use by others?

Yes

Reviewer Expertise:

Medical Education, Forensic Medicine and Clinical Toxicology

I confirm that I have read this submission and believe that I have an appropriate level of expertise to confirm that it is of an acceptable scientific standard, however I have significant reservations, as outlined above.

# Peer review: Reviewer response for version 2

**DOI:** 10.21956/mep.20511.r31937

**Published:** 2022-05-17

**Authors:** Helen Atherton

**Affiliations:** 1 Unit of Academic Primary Care, University of Warwick, Coventry, UK

I am happy with the changes.

Is the rationale for developing the new method (or application) clearly explained?

Partly

Is the description of the method technically sound?

No

Are the conclusions about the method and its performance adequately supported by the findings presented in the article?

Partly

If any results are presented, are all the source data underlying the results available to ensure full reproducibility?

Partly

Are sufficient details provided to allow replication of the method development and its use by others?

No

Reviewer Expertise:

General practice, the consultation, access to general practice, digital health

I confirm that I have read this submission and believe that I have an appropriate level of expertise to confirm that it is of an acceptable scientific standard.

# Peer review: Reviewer response for version 1

**DOI:** 10.21956/mep.18786.r31803

**Published:** 2022-04-11

**Authors:** Chris Roberts

**Affiliations:** 1 Health Professions Education, Sydney Medical School, University of Sydney, Sydney, NSW, Australia

I very much enjoyed reading and reading this paper describing some early validation evidence for the use of a general practice video consultation rating scale (DanOBS). The scale was developed in the context of an immersive general practice placement for senior medical students. (I didn’t easily spot what DanOBS stands for as an acronym).

 As I understand it, the authors experienced some shortcomings with existing tools when considering the performance of students in patient centred consultations, and the time available for rating. They developed a multi-step process of reviewing the literature and consulting with students and teaching faculty to come up with a new or modified scale of 13 items (DanOBS). Having developed the prototype they then went on to collect some further validity evidence on its usage from 79% of the cohort (n= 295/375) including test retest reliability.  It was used as an assessment of student performance. This was done by testing students on a video pre-course and then post-course. They then go on to recommend contexts where the tool might be used as a way of rating a clinical tutors’ performance. 

 There is much to commend in this work, and the DanOBS looks like a useful (and short tool) to rate student performance either formatively or as part of a summative assessment of the placement with useful checklist items to provide qualitative feedback to the student.  Others in GP settings may find that they can adapt this scale to their local context. I am less convinced in its current version that it could be used to evaluate teaching quality.  In developing this work in the future, the authors might like to consider other examples of the reporting of validity evidence for scale development for use in general practice as a teaching or assessment tool. Whilst the content and process aspects of the tool is well described, demonstrating the internal structure of DanOBS might have used Cronbach’s for internal consistency, and a factor analysis to determine the number of domains in the scale. It might be interesting to see how the total score relates to student performance? The test retest might not be your strongest validity evidence?

 I think writing upscale development is hard to do and a graphic/table of the differing steps, including the assessment involved in the process, might help.  My suggestions for strengthening the article relate to

1. Making a strong case for why a new scale is required
2. Clarifying each of the steps of instrument development, so that someone else reading this article can exactly follow the steps and come up with similar results.
3. Providing as much detail of the assessment as possible to judge the psychometric claims
4. Reconsider the conclusion about teacher evaluation based on the data presented in this study.

I would encourage the authors to further develop the validity evidence for this scale. Given how much work new scale development involves, I would suggest taking some advice from the psychometricians on current ways of reporting validity evidence.

 Is the version DanOBS questionnaire 2.docx the version you would wish others to use in their own research contexts?  

Is the rationale for developing the new method (or application) clearly explained?

Partly

Is the description of the method technically sound?

Partly

Are the conclusions about the method and its performance adequately supported by the findings presented in the article?

Partly

If any results are presented, are all the source data underlying the results available to ensure full reproducibility?

Yes

Are sufficient details provided to allow replication of the method development and its use by others?

No

Reviewer Expertise:

Professionalism, assessment, community based education

I confirm that I have read this submission and believe that I have an appropriate level of expertise to confirm that it is of an acceptable scientific standard, however I have significant reservations, as outlined above.

# Response

**Published:** 2022-04-20

**Authors:** Merete Jorgensen

**Affiliations:** Copenhagen University, Denmark

Thanks a lot for your comment. The tool can be used before and after courses to measure teaching effectiveness. It can be used as a training tool for students, but not for summative assessments.   I see your point about the validity testing and find a need to clarify this issue in the next version, but here is a short answer: As I write in the article no constructs, especially for medical students observing a consultation exist. As for consistency, it can be difficult as the items in the different domains are very different. But I will work on an understandable comment. Volkert Siersma is a statistician who works with psychometrics and he gives the explanation above. We will make it more clear in the next version.

# Peer review: Reviewer response for version 1

**DOI:** 10.21956/mep.18786.r31740

**Published:** 2022-03-29

**Authors:** Helen Atherton

**Affiliations:** 1 Unit of Academic Primary Care, University of Warwick, Coventry, UK

This is a really interesting report about an approach to assessing the patient centred consultation model in medical schools doing general practice placements. The team have used the resources at their disposal to come up with a tool to apply in their setting. 

 Whilst an interesting report there were some areas that it would be helpful to have more information:

- In the methods, under 'first step' please can you provide more information about the process of selection? Was it simply preference or was there a methodological approach taken?
- In the methods under 'third step' please can you clarify whether the video consultation watched is a consult carried out by the student or is an example consult? Are these consultations face to face consultations that are video taped or are they remote consultations? Denmark uses a lot of email consultation, does it apply to those too? 
- For the 'modification of the response options' how were these decisions made? Was it again down to personal preference or was it based on evidence about how people answer these sorts of things? 
- Can you justify the use of students to conduct this work? Why not use a mixture of participants including healthcare professionals? The use of volunteers to refine the tool is a significant limitation and this should be mentioned.  Overall the limitations are not really discussed and it would add to this work if you were explicit in the discussion about the issues with the methodology so that future studies could plan to avoid these. 
- Since conducting this work the number of remote consultations has increased. Although your work does seemingly not cover this, it would help to see some comment in the discussion about how this tool would apply to a remote consultation (telephone, video, email)? 
- The conclusion refers to the tool being used to inform the teachers about the effectiveness of their teaching. Why isn't it a tool to inform the students about the effectiveness of their learning? Unfortunately, I don't see how this tool can claim to assess effectiveness in either direction when it has been devised 'in house' and with volunteers. It would be better to describe it as a tool that can guide learning in students in this setting, and that is open to future validation in rigorous independent studies. 

This article is a useful description that other providers may wish to draw on in devising their own approaches that work for their own setting and if desired with further work this could be developed further, though this is not necessary for setting specific use.

Is the rationale for developing the new method (or application) clearly explained?

Partly

Is the description of the method technically sound?

No

Are the conclusions about the method and its performance adequately supported by the findings presented in the article?

Partly

If any results are presented, are all the source data underlying the results available to ensure full reproducibility?

Partly

Are sufficient details provided to allow replication of the method development and its use by others?

No

Reviewer Expertise:

General practice, the consultation, access to general practice, digital health

I confirm that I have read this submission and believe that I have an appropriate level of expertise to confirm that it is of an acceptable scientific standard, however I have significant reservations, as outlined above.

# Response

**Published:** 2022-04-07

**Authors:** Merete Jorgensen

**Affiliations:** Copenhagen University, Denmark

Thanks a lot for your comments which I find helpful and I will comment and revise the article accordingly. The test for face and content validity was performed with medical students and cannot easily be tansferred to other health personal.
